# *Salmonella* Rapidly Regulates Membrane Permeability To Survive Oxidative Stress

**DOI:** 10.1128/mBio.01238-16

**Published:** 2016-08-09

**Authors:** Joris van der Heijden, Lisa A. Reynolds, Wanyin Deng, Allan Mills, Roland Scholz, Koshi Imami, Leonard J. Foster, Franck Duong, B. Brett Finlay

**Affiliations:** a Michael Smith Laboratories, University of British Columbia, Vancouver, British Columbia, Canada; b Department of Microbiology and Immunology, University of British Columbia, Vancouver, British Columbia, Canada; c Department of Biochemistry and Molecular Biology, University of British Columbia, Vancouver, British Columbia, Canada; d Center for High-Throughput Biology, University of British Columbia, Vancouver, British Columbia, Canada

## Abstract

The outer membrane (OM) of Gram-negative bacteria provides protection against toxic molecules, including reactive oxygen species (ROS). Decreased OM permeability can promote bacterial survival under harsh circumstances and protects against antibiotics. To better understand the regulation of OM permeability, we studied the real-time influx of hydrogen peroxide in *Salmonella* bacteria and discovered two novel mechanisms by which they rapidly control OM permeability. We found that pores in two major OM proteins, OmpA and OmpC, could be rapidly opened or closed when oxidative stress is encountered and that the underlying mechanisms rely on the formation of disulfide bonds in the periplasmic domain of OmpA and TrxA, respectively. Additionally, we found that a *Salmonella* mutant showing increased OM permeability was killed more effectively by treatment with antibiotics. Together, these results demonstrate that Gram-negative bacteria regulate the influx of ROS for defense against oxidative stress and reveal novel targets that can be therapeutically targeted to increase bacterial killing by conventional antibiotics.

## IMPORTANCE

Pathogenic bacteria have evolved ways to circumvent inflammatory immune responses. A decrease in bacterial outer membrane permeability during infection helps protect bacteria from toxic molecules produced by the host immune system and allows for effective colonization of the host. In this report, we reveal molecular mechanisms that rapidly alter outer membrane pores and their permeability in response to hydrogen peroxide and oxidative stress. These mechanisms are the first examples of pores that are rapidly opened or closed in response to reactive oxygen species. Moreover, one of these mechanisms can be targeted to artificially increase membrane permeability and thereby increase bacterial killing by the antibiotic cefotaxime during *in vitro* experiments and in a mouse model of infection. We envision that a better understanding of the regulation of membrane permeability will lead to new targets and treatment options for multidrug-resistant infections.

## INTRODUCTION

During infection with bacterial pathogens, the immune response of healthy individuals generates antimicrobial reactive oxygen species (ROS) to kill invading bacteria. The outer membrane (OM) of Gram-negative bacterial pathogens provides protection from a variety of environmental stresses, including ROS (1). ROS can permeate through the bacterial membrane to cause damage to bacterial proteins, DNA, and other intrabacterial molecules (2, 3). However, very little is known about if or how bacteria regulate the influx of ROS. The influx of antibiotics has been more extensively studied (1, 4). Typically, hydrophobic compounds diffuse through the OM while hydrophilic molecules permeate into bacteria predominantly through pores in OM proteins (OMPs) (1). Since many antibiotics enter through OM pores, accurate regulation of OMP expression lies at the core of antibiotic resistance, which is clearly illustrated by a decreased OM permeability in a majority of the multidrug-resistant bacteria isolated from patients in clinics (5, 6). Recent studies have revealed that there are subsets of pathogens in different tissue microenvironments with disparate outcomes of antibiotic treatment (7). Certain subsets include nonreplicating antibiotic-tolerant cells that limit OM permeability and often reinitiate a full-blown infection after antibiotic treatment is completed (1, 8–13). Because of this, it has been suggested that targeting the OM to increase permeability is an underexploited strategy to increase antibiotic efficacy (14). Recently, we described an analytical method that relies on redox-sensitive green fluorescent protein (GFP), called roGFP2, to measure redox changes directly inside bacteria, enabling us to measure the real-time influx of ROS (15, 16). In this study, we used this system to accurately measure the real-time influx of H_2_O_2_ into living *Salmonella enterica* serovar Typhimurium bacteria during exposure to ROS and identified regulatory mechanisms that alter OM permeability and ROS sensitivity.

## RESULTS

### Pores in OMPs facilitate H_2_O_2_ diffusion across the OM and control its permeability.

**FIG 1:**
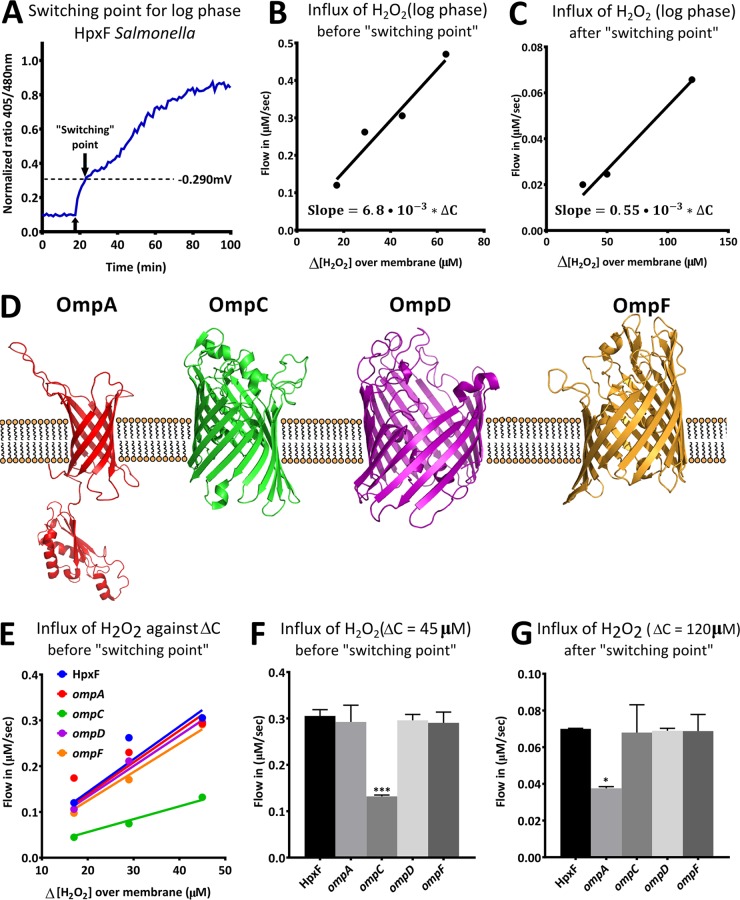
*Salmonella* rapidly reduces OM permeability at the switching point. (A) Real-time analysis of changes to the intrabacterial H_2_O_2_ concentration after a challenge with 150 µM H_2_O_2_. The upward-pointing arrow indicates injection of H_2_O_2_. The downward-pointing arrow indicates the moment after which the H_2_O_2_ influx was suddenly reduced. We termed this moment the switching point. (B, C) Correlation between the H_2_O_2_ influx across the *S*. Typhimurium membrane and Δ*C* before (B) and after (C) the switching point. (D) Schematic illustration of four OMPs in the OM. The protein structures of OmpA, OmpC, OmpF, and the periplasmic domain of OmpA were taken from http://www.wwpdb.org/. The structural prediction of OmpD was performed with the iTasser server at http://zhanglab.ccmb.med.umich.edu/I-TASSER/. (E) For each of the single OMP deletion mutants, a correlation between the H_2_O_2_ influx and Δ*C* was obtained before the switching point. (F, G) H_2_O_2_ influx levels were obtained before and after the switching point at Δ*C* = 45 µM and Δ*C* = 120 µM, respectively. All experiments were done with mutants in the HpxF^−^ background. Each value represents the average of four separate experiments. Error bars represent the standard deviation, and statistical significance was determined by one-way analysis of variance with comparison to the HpxF^−^ group (*, *P* < 0.05; ***, *P* < 0.001).

It is often assumed that H_2_O_2_ can freely cross membranes. However, several studies show that certain membranes are poorly permeable to H_2_O_2_ (3, 17–19). In these membranes, permeability can be regulated by changes in membrane lipid composition or by diffusion-facilitating channel proteins. To study membrane permeability for H_2_O_2_ in Gram-negative bacteria, we measured real-time H_2_O_2_ influx by utilizing roGFP2 in the HpxF^−^ background of *S*. Typhimurium. This particular strain is devoid of catalases and peroxidases (20) so that rapid detoxification of intrabacterial H_2_O_2_ could be avoided and additionally so that biologically relevant concentrations of H_2_O_2_ could be investigated (16). The detoxifying power of the HpxF^−^ mutant was ~90-fold lower than that of wild-type (WT) *S*. Typhimurium, and exposure to increasing amounts of H_2_O_2_ led to a dose-dependent response (see Fig. S1A to C in the supplemental material). After real-time monitoring of the H_2_O_2_ influx in log-phase bacteria, we found that H_2_O_2_ diffuses passively over the membrane in the initial period following H_2_O_2_ exposure (Fig. 1A). However, we observed a previously unreported rapid reduction in H_2_O_2_ influx coinciding with an intrabacterial redox potential of about −290 mV (Fig. 1A). To more accurately investigate the details of what we termed the “switching point,” we used the equation Influx = *P ⋅ A ⋅* Δ*C*, which defines the correlation between influx and membrane permeability for passive diffusion (3).

**FIG 2:**
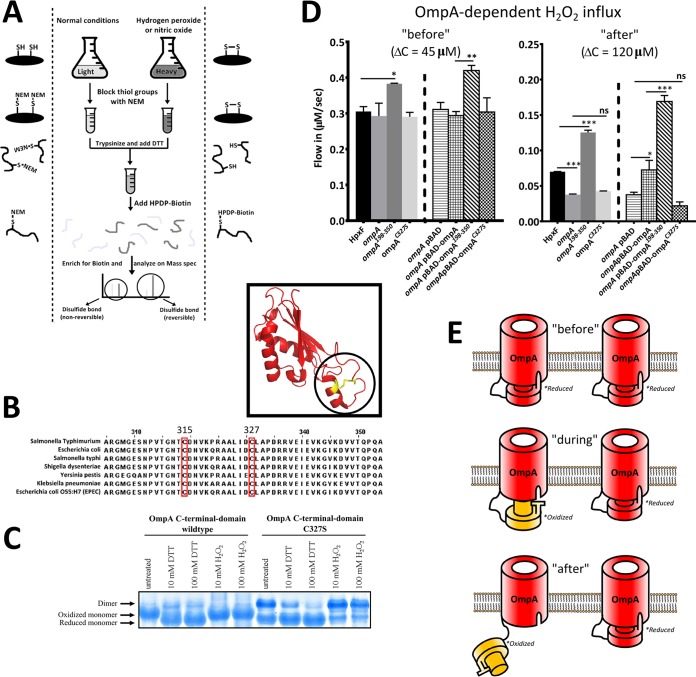
Opening of the OmpA pore is regulated by its periplasmic domain. (A) SILAC mass spectrometry proteomics procedure aimed at identifying reversible disulfide bonds in the *S*. Typhimurium proteome. Bacterial cultures were grown for 5 h while their proteome was labeled with light or heavy amino acids. After exposure to 10 mM H_2_O_2_ or 10 mM spermine NONOate (nitric oxide), the bacteria were lysed, the reversible disulfides were labeled with HPDP-biotin {*N*-[6-(biotinamido)hexyl]-3′-(2′-pyridyldithio)propionamide}, and the peptides originating from the oxidized culture were compared to peptides originating from the control (normoxic) culture by mass spectrometry. (B) Sequence comparison shows that the two cysteines in the periplasmic domain of OmpA are widely conserved among many Gram-negative bacteria. (C) Native acrylamide gel showing the purified periplasmic domains of WT OmpA and mutant OmpA^C327S^ after these were subjected to various concentrations of DTT or H_2_O_2_. The conformational change that is observed for the monomer under oxidizing conditions are visible only in lanes containing the WT periplasmic domain. It is worth noting that untreated purified proteins automatically get oxidized over time if no reducing agent is added. (D) The influx of H_2_O_2_ was determined before and after the switching point. The H_2_O_2_ influx levels before and after the switching point were determined at Δ*C* = 45 µM and Δ*C* = 120 µM, respectively. Before the switching point, the influx in the *ompA*^188-350^ mutant was significantly higher than the influx in HpxF^−^ *S*. Typhimurium. After the switching point, the influx in the *ompA* or *ompA*^C327S^ mutant (which is unable to form the reversible disulfide bond) was lower than the influx in HpxF^−^ *S*. Typhimurium. No significant difference was found between the *ompA* and *ompA*^C327S^ mutant influx levels*.* The influx in the *ompA*^188-350^ mutant was significantly higher than the influx in HpxF^−^ *S*. Typhimurium. Complementation of *ompA* with the arabinose-inducible pBAD plasmid containing *ompA*, *ompA*^198-350^, or *ompA*^C327S^ confirmed the results obtained with genomic mutations. All experiments were done with mutants in the HpxF^−^ background. Each value represents the average of four separate experiments. Error bars represent the standard deviation, and statistical significance was determined by one-way analysis of variance with comparison to the HpxF^−^ group (*, *P* < 0.05; **, *P* < 0.01; ***, *P* < 0.001; ns, not significant). (E) Schematic representation of a proposed mechanism that leads to opening and closing of the OmpA pore by its periplasmic domain. Formation of the disulfide bond at the switching point regulates opening of the pore.

In this equation, *P* is the membrane permeability coefficient, *A* is the surface area of the bacterial membrane (which we assumed to be constant over short measurements), and Δ*C* is the difference between the H_2_O_2_ concentrations on both sides of the membrane. The influx over a variety of different Δ*C* values was measured, and before the switching point, we found a linear correlation between influx (µM/s) and Δ*C*, which is consistent with the rules for passive diffusion (Fig. 2B and C). However, when analyzing influx after the switching point for equal Δ*C* values, we found that influx was reduced to only ~8% of the influx before this point. Since the total bacterial surface area is constant throughout the experiment, this reduction can only be explained by rapid alterations in membrane permeability. A similar analysis of stationary-phase bacteria also revealed a switching point that led to a reduction in membrane permeability in which the remaining permeability was only ~3% of the permeability before the switching point (see Fig. S1D to F in the supplemental material). The change in membrane permeability was too fast to be facilitated by altered lipid composition, and on the basis of these findings, we hypothesized that at least part of the H_2_O_2_ diffusion was facilitated by pores or channels in the OM. To verify that changes in the OM were driving the rapid alteration in membrane permeability, the OM was removed and the H_2_O_2_ influx in spheroplasts was measured (see Fig. S1G in the supplemental material). In spheroplasts, no switching point was observed, indicating that changes to the OM were responsible for the rapid reduction of membrane permeability. Since the influx in spheroplasts was calculated to increase to ~350% of the H_2_O_2_ influx in intact bacteria, we conclude that OM permeability is the rate-limiting determinant of overall membrane permeability. This is in agreement with previous findings obtained with other bacteria, where, because of lipid composition, the rate of diffusion over the inner membrane is expected to be orders of magnitude greater than that over the OM (21).

Several OMPs form a beta-barrel in the OM and can potentially facilitate the diffusion of hydrophilic molecules across the OM (Fig. 1D) (22). To identify the OMPs responsible for H_2_O_2_ diffusion, we created single deletion mutants of the four most abundant OMPs (OmpA, OmpC, OmpD, and OmpF) in HpxF^−^ *S*. Typhimurium and calculated the H_2_O_2_ influx for increasing Δ*C* values before the switching point for each of the mutants (Fig. 1E). For easier comparisons, subsequent analysis of the influx throughout this study was done at a Δ*C* of 45 µM (before the switching point) and a Δ*C* of 120 µM (after the switching point). Our results indicated that OmpC predominantly facilitated H_2_O_2_ diffusion before the switching point (Fig. 1E and F), whereas OmpA facilitated H_2_O_2_ diffusion after the switching point (Fig. 1G). Under the conditions used in this study, OmpD and OmpF did not contribute to the diffusion of H_2_O_2_ in *S*. Typhimurium. None of the OMPs completely abrogated membrane diffusion of H_2_O_2_, suggesting that low levels of H_2_O_2_ diffuse freely across the membrane.

### Opening of the OmpA pore is controlled by its periplasmic domain and is dependent on oxidation-sensitive cysteines.

Our findings indicate rapid switching from the OmpC pore to the OmpA pore to facilitate H_2_O_2_ influx at increasing levels of oxidative stress. Additionally, these results indicate that these pores can be rapidly closed or opened when oxidative stress is encountered. Interestingly, the OmpA protein differs from most other OMPs by having an extensive periplasmic domain (Fig. 1D). A key feature of this periplasmic domain is that it harbors two cysteines that have been shown to form an internal disulfide bond (23). Certain disulfide bonds have been found to depend on oxidative stress and can thereby function as a regulatory switch (24). To investigate the potential reversible nature of this OmpA-disulfide bond, we performed a mass spectrometry experiment that utilized stable isotope labeling by amino acids (SILAC) to compare disulfide bond formation in the *S*. Typhimurium proteome under oxidizing and normoxic conditions (Fig. 2A). We found that the disulfide bond between the only two cysteines in the periplasmic domain of OmpA occurred preferentially under oxidizing conditions (see Table S1 in the supplemental material). An additional genetic comparison found that these two cysteines are conserved in the periplasmic domain of OmpA in several Gram-negative bacteria (Fig. 2B). On the basis of this information, we hypothesized that the periplasmic domain of OmpA could function as a plug domain, while formation of the reversible disulfide bond could be the regulatory switch that controlled the conformation of the periplasmic domain and thereby the opening and closing of the OmpA pore. To investigate this concept, we first purified the WT periplasmic domain of OmpA (amino acids 198 to 350) and a mutant periplasmic domain that is unable to form an internal disulfide bond (cysteine 327 was replaced with a serine residue). These two purified proteins were subjected to oxidizing or reducing conditions (various concentrations of H_2_O_2_ or dithiothreitol [DTT], respectively) and loaded onto a native acrylamide gel. We found that the WT OmpA periplasmic domain undergoes a conformational change upon oxidation, whereas the OmpA^C327S^ mutant periplasmic domain does not change its conformation (Fig. 2C). Furthermore, we created a deletion mutant of the HpxF^−^ strain that lacked the periplasmic domain (*ompA*^198-350^ mutant) and a point mutant (*ompA*^C327S^ mutant) that is unable to form an internal disulfide bond and measured the H_2_O_2_ influx before and after the switching point (Fig. 2D). Before the switching point, when our data suggest that the OmpA pore is closed, deletion of the periplasmic domain resulted in increased H_2_O_2_ influx, suggesting permanent opening of the pore. After the switching point, when our data indicate that the OmpA pore is opened, deletion of *ompA* and abrogation of disulfide bond formation in *ompA*^C327S^ both resulted in decreased OM permeability. Under these circumstances, deletion of the periplasmic domain resulted in increased OM permeability, indicating that in WT bacteria, even after the switching point, not all OmpA pores are opened under our experimental conditions. Complementation of the *ompA* mutant with the arabinose-inducible pBAD plasmid containing *ompA*, *ompA*^198-350^, or *ompA*^C327S^ confirmed the specific involvement of OmpA and its periplasmic domain in the regulation of H_2_O_2_ influx after the switching point (Fig. 2D). These results support the concepts that the periplasmic domain of OmpA functions as a plug domain and that reversible disulfide bond formation in this periplasmic domain regulates the opening and closing of the pore. A schematic representation of the proposed mechanism is shown in Fig. 2E.

### The OmpC pore relies on the proteins HslT and TrxA for H_2_O_2_ diffusion.

**FIG 3:**
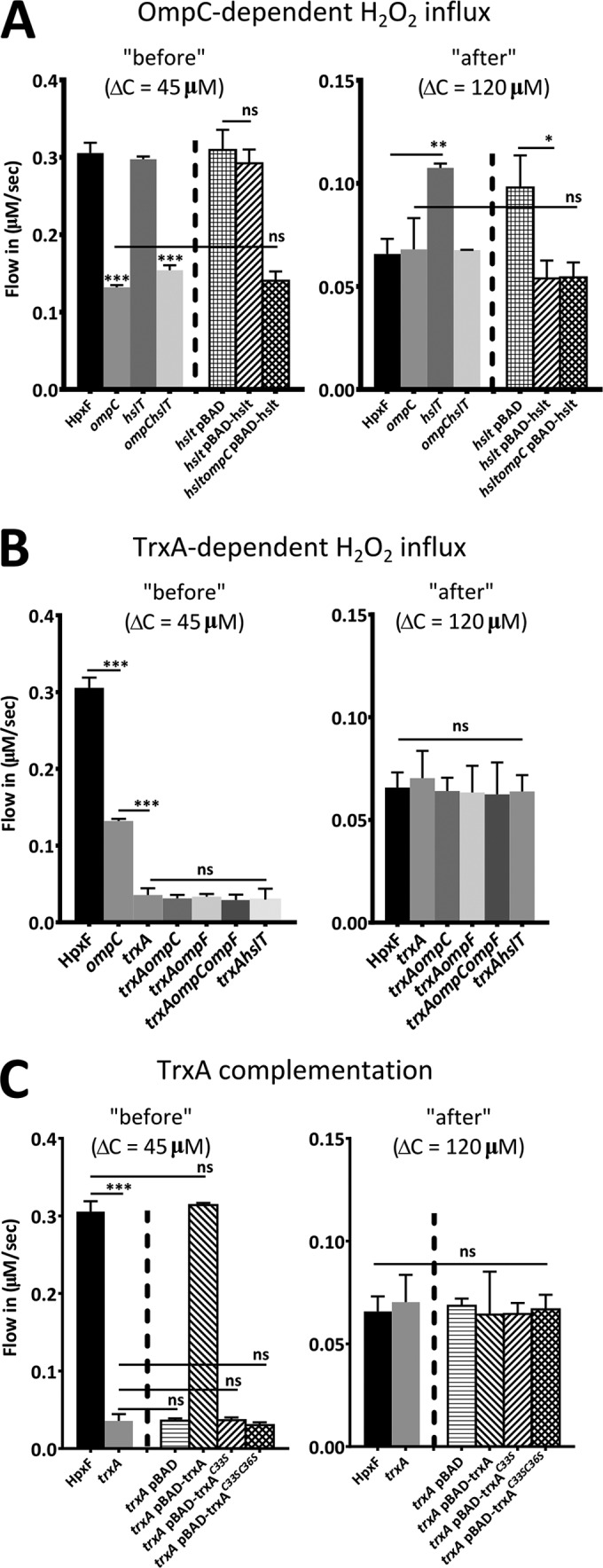
HslT and TrxA are required for H_2_O_2_ influx through the OmpC pore. (A) H_2_O_2_ influx into *Salmonella*. H_2_O_2_ influx levels before and after the switching point were determined at Δ*C* = 45 µM and Δ*C* = 120 µM, respectively. Before the switching point, the influx in *ompC* and *ompC hslT* mutant bacteria was significantly lower than the influx in HpxF^−^ *S*. Typhimurium. After the switching point, the influx in *hslT* mutant bacteria was significantly greater than the influx in HpxF^−^ *S*. Typhimurium. Complementation of *hslT* or *hslT ompC* with the arabinose-inducible pBAD plasmid containing the *hslT* gene reverted the phenotype observed in the *hslT* mutant background. (B) Influx of H_2_O_2_ into *Salmonella* before and after the switching point. Deletion of *trxA* decreases H_2_O_2_ influx before the switching point. Additional deletions of *ompC*, *ompF*, *ompC ompF*, and *hslT* do not further decrease H_2_O_2_ influx before the switching point. (C) Complementation of *trxA* with the arabinose-inducible pBAD plasmid containing the *trxA* gene or the mutant *trxA*^C33S^ or *trxA*^C33SC36S^ gene. Complementation with WT *trxA* reverts the decreased H_2_O_2_ influx to WT levels; however, complementation with mutant *trxA* genes does not revert the decrease in membrane permeability. Each value represents the average of four separate experiments. All experiments were done with mutants in the HpxF^−^ background. Error bars represent the standard deviation, and statistical significance was determined by one-way analysis of variance with comparison to the HpxF^−^ group (*, *P* < 0.05; **, *P* < 0.01; ***, *P* < 0.001; ns, not significant).

In contrast to the OmpA pore, the OmpC pore appeared to be opened before and closed after the switching point (Fig. 1E to G). Since the OmpC protein does not contain a periplasmic domain, we hypothesized that other periplasmic proteins could be involved in the regulation of the opening and closing of the OmpC pore. Previous studies have identified binding partners for OmpC and shown that in *Escherichia coli*, small heat shock protein A (IbpA) interacts with the OmpC protein (25). IbpA has also been found to be associated with the OM and the periplasm (26). IbpA is homologous to *Salmonella* protein HslT, with 98% sequence similarity. To determine whether HslT could be involved in the regulation of H_2_O_2_ influx through the OmpC pore, we measured H_2_O_2_ influx before and after the switching point in the *ompC* and *hslT* single deletion mutants and the *hslT ompC* double mutant (Fig. 3A). Before the switching point, when our data suggest that the OmpC pore is open, H_2_O_2_ influx was lower in the *ompC* and *hslT ompC* mutants. After the switching point, when our findings suggest that the OmpC pore is closed, deletion of *hslT* increased H_2_O_2_ influx. Influx was restored to WT levels in the *hslT ompC* mutant, indicating that increased influx in the absence of *hslT* was dependent on the presence of OmpC. Results from complementation of the *hslT* or *hslT ompC* mutant with the pBAD-hslT plasmid showed that increased membrane permeability in *hslT* after the switching point is specific for HslT and relies on the presence of OmpC (Fig. 3A). It is likely that other pores are involved in the control of H_2_O_2_ diffusion, since an *hslT* deletion does not revert H_2_O_2_ influx back to the influx that was measured before the switching point.

In addition to HslT, the OmpC pore has been found to interact with the oxidation-sensitive protein thioredoxin (TrxA) (27). After measuring the H_2_O_2_ influx in a *trxA* mutant, we found that the H_2_O_2_ influx before the switching point was dramatically lower in the mutant than in WT bacteria (Fig. 3B). After the switching point, no change in H_2_O_2_ influx was observed. Deletion of *trxA* in combination with *ompC*, *ompF*, and/or *hslT*, did not result in further increased or decreased membrane permeability, suggesting that TrxA is required for H_2_O_2_ diffusion through the OmpC pore (Fig. 3B). Since membrane permeability in a *trxA* mutant was lower than that in an *ompC* single mutant, we speculate that TrxA also aids other mechanisms of H_2_O_2_ transport across the OM that were not investigated in this study. Several studies have found that TrxA forms an internal reversible disulfide bond similar to the reversible disulfide bond in the OmpA periplasmic domain (28). Transformation of *trxA* with pBAD plasmids containing either WT *trxA* or the *trxA*^C33S^ or *trxA*^C33SC36S^ mutant gene, which is defective in disulfide bond formation, showed that decreased membrane permeability could be reversed by complementation with *trxA* but not by complementation with either of the mutant *trxA* genes (Fig. 3C). These results suggest that TrxA and its reversible disulfide bond are required for H_2_O_2_ diffusion prior to the switching point.

To control for differential expression of OMPs under mutant conditions, we tested the expression of OmpA in different mutants (see Fig. S2 in the supplemental material). No differences in the level of expression were observed. On the basis of these results, it is unlikely that differential expression of OMPs is the main reason for our observations.

### Experimentally increased OM permeability aids bacterial killing.

**FIG 4:**
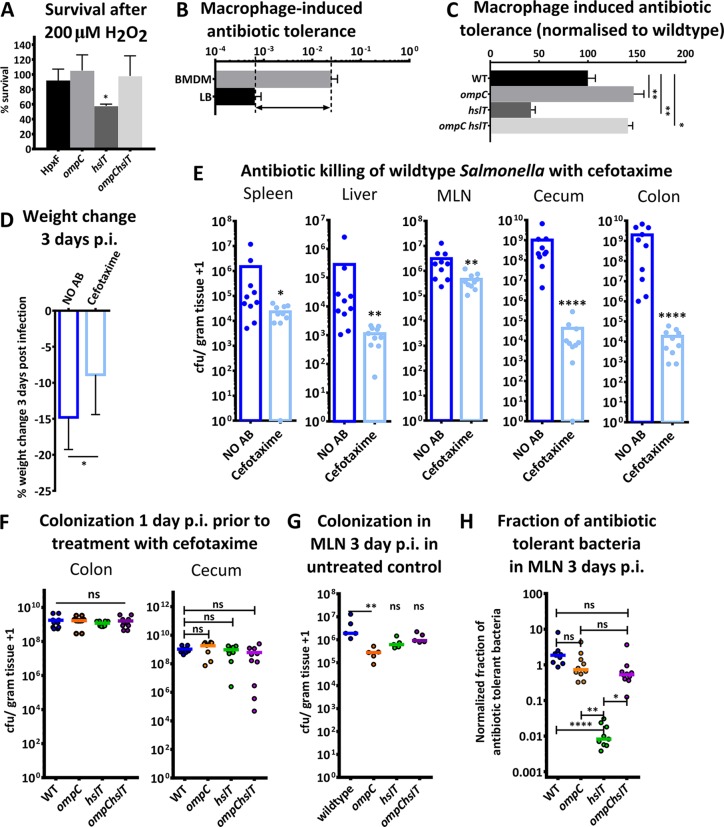
Increased OM permeability increases antibiotic efficacy. (A) Percentages of HpxF^−^, *ompC*, *hslT*, and *ompC hslT* mutant bacteria that survived exposure to 200 µM H_2_O_2_ for 2 h. These experiments were done with bacteria in the HpxF^−^ background. (B) Thirty minutes after BMDM internalization, bacteria were recovered and treated with 100 µg/ml cefotaxime for 24 h in LB. CFU counts were significantly higher on plates with *S*. Typhimurium bacteria that had interacted with BMDM. (C) Numbers of antibiotic-tolerant WT and *ompC*, *hslT*, and *ompC hslT* mutant bacteria after 24 h in LB medium supplemented with 100 µg/ml cefotaxime. For each mutant, equal numbers of bacteria that were retrieved from BMDM were analyzed during antibiotic treatment. Antibiotic tolerance was analyzed by *Salmonella* CFU counting. Error bars represent the standard deviation, and significance was obtained by one-way analysis of variance. (D) The body weight change of mice 3 days postinfection after treatment with saline (NO AB) or cefotaxime for 2 days beginning 1 day postinfection. Body weight change is presented as a percentage of the body weight prior to infection. (E) CFU counts of *S*. Typhimurium bacteria from the spleen, liver, MLN, cecum, and colon 3 days postinfection with or without a 2-day treatment with cefotaxime. Significantly fewer bacteria were found in each organ after antibiotic treatment. Statistical significance was determined by a Mann-Whitney test. (F) CFU counts of WT and *ompC*, *hslT*, and *ompC hslT* mutant bacteria from the colons and ceca of mice at 1 day postinfection, prior to cefotaxime treatment. No significant colonization differences from WT bacteria were observed. (G) CFU counts of WT and *ompC*, *hslT*, and *ompC hslT* mutant bacteria in the MLN at 3 days postinfection without antibiotic treatment. Only *ompC* mutant bacteria showed a slightly attenuated ability to colonize the MLN. (H) Normalized fraction of bacteria that shows antibiotic tolerance in the MLN after treatment with cefotaxime. The fraction of antibiotic-tolerant bacteria after cefotaxime treatment was normalized by using the median number of the corresponding mutant bacteria that colonized the MLN of untreated mice. All experiments were done with mutants in the WT background. Statistical significance was determined by a Kruskal-Wallis test with comparison to the WT control group (*, *P* < 0.05; **, *P* < 0.01; ****, *P* < 0.0001; ns, not significant).

To our knowledge, this is the first report describing how Gram-negative bacteria rapidly control OM permeability by closing or opening pores in OMPs. To examine the clinical relevance of our findings, we assessed whether the survival of bacteria under oxidizing conditions was impaired by experimentally increased OM permeability. For this, we measured the survival of WT and *ompC*, *hslT*, and *ompC hslT* mutant bacteria after a challenge with 200 µM H_2_O_2_ (Fig. 4A). Only the *hslT* mutant, which was found to have increased OM permeability, was attenuated for survival, while mutants with decreased OM permeability did not seem to be compromised. These results show that an inability to lower OM permeability impairs bacterial survival under oxidizing conditions.

We speculated that increasing OM permeability would increase antibiotic efficacy and allow for better bacterial killing. To test this hypothesis, we used our mutants with variable levels of OM permeability and tested the number of bacteria that survived exposure to cefotaxime. According to a recent publication, a brief interaction with bone marrow-derived macrophages (BMDM) prior to antibiotic exposure can be used to increase the number of antibiotic-tolerant bacteria (10). We used this method and found that after a 30-min macrophage interaction, a greater subset of bacteria survived exposure to cefotaxime (Fig. 4B). Since the OmpC pore is larger than the OmpA pore and could therefore facilitate the diffusion of antibiotics, we quantified the numbers of surviving WT and *ompC*, *hslT*, and *ompC hslT* mutant *S*. Typhimurium bacteria after exposure to cefotaxime (Fig. 4C). It has been shown that ROS are not the driving force behind the phenotypic transformation into antibiotic-tolerant bacteria during macrophage interaction (10). Instead, macrophage interaction is merely used to increase the numbers of antibiotic-tolerant bacteria prior to antibiotic exposure. Importantly, the entire antibiotic exposure and the quantification of surviving bacteria took place *in vitro* without the presence of macrophages. The numbers of surviving bacteria were normalized to the numbers of bacteria at the start of the experiment, thereby controlling for differences in macrophage uptake and differences in growth/killing by macrophage-inflicted defense. Interestingly, we found that killing by cefotaxime was less effective in mutants with decreased OM permeability (*ompC* and *ompC hslT*), while antibiotic efficacy was greater in the *hslT* mutant (with increased OM permeability) than in WT bacteria (Fig. 4C). As a control, we determined the MICs of cefotaxime for WT or *hslT*, *ompC*, or *ompC hslT* mutant bacteria and found no significant differences (data not shown). Together, these results support the hypothesis that increased OM permeability increases antibiotic efficacy by killing a subset of the antibiotic-tolerant cells.

To further investigate this concept, we examined whether *S*. Typhimurium would survive cefotaxime treatment in a mouse model of infection. Previously, cefotaxime has been found to kill extracellular and intracellular bacteria equally effectively, which excludes potential differences because of differential cellular internalization of *S*. Typhimurium (29). We utilized an infection protocol similar to that described in a recent study showing that a subset of *Salmonella* bacteria survives antibiotic treatment of mice (11). We infected mice with *S*. Typhimurium and at 1 day postinfection began treating them with cefotaxime for 2 days. After antibiotic treatment, we found that mice still lost weight and carried antibiotic-tolerant bacteria in the spleen, liver, mesenteric lymph nodes (MLN), cecum, and colon (Fig. 4D and E). We detected the highest numbers of antibiotic-tolerant bacteria in MLN samples after cefotaxime treatment (Fig. 4E), similar to previous findings that reported the MLN as a reservoir of persister bacteria (11).

To test whether increased OM permeability would increase antibiotic efficacy, we infected mice with WT, *ompC* mutant (decreased OM permeability), *hslT* mutant (increased OM permeability), or *ompC hslT* mutant (decreased OM permeability) *S*. Typhimurium. A complicating factor in assessing the effect of experimentally increased OM permeability on antibiotic killing during mouse infections is the side effect that alterations in OM permeability sometimes lead to some attenuation of bacterial fitness/virulence. We first determined that, at day 1 postinfection (the commencement of antibiotic treatment), no significant differences in colonization of the cecum and colon existed between WT bacteria and any of the mutants (Fig. 4F). At day 1 postinfection, we found no significant colonization of systemic organs yet. To investigate the subset of antibiotic-tolerant bacteria, we focused on the MLN as the main reservoir after antibiotic treatment. In the untreated control group at 3 days postinfection, only the *ompC* single mutant appeared to have a slightly attenuated ability to colonize the MLN (Fig. 4G). Both the *hslT* and *ompC hslT* mutants colonized the MLN to a level similar to that of the WT bacteria. In order to control for differences in the ability to colonize the MLN, the number of bacteria that survived treatment with cefotaxime was normalized to the median number of colonizing bacteria for each of the mutants (Fig. 4H). We found that cefotaxime treatment killed significantly more *hslT* mutant than WT *S*. Typhimurium bacteria in the MLN. No significant differences were observed for *ompC* or *ompC hslT* mutant bacteria, suggesting that experimentally increased OM permeability was most likely the reason for increased antibiotic efficacy. Additional analysis of WT and *ompC*, *hslT*, and *ompC hslT* mutant bacteria at 3 days postinfection without antibiotic treatment revealed that *ompC* mutant *S*. Typhimurium bacteria have an especially attenuated ability to colonize systemic organs (see Fig. S3A in the supplemental material). Despite this apparent disadvantage, greater numbers of *ompC* mutant bacteria (decreased OM permeability) than *hslT* mutant bacteria (increased OM permeability) survived in the MLN after treatment with cefotaxime (see Fig. S3B). Together, these results suggest that artificially increasing OM permeability is an effective way of increasing antibiotic efficacy.

## DISCUSSION

In this report, we reveal a novel stress response mechanism by which *Salmonella* regulates OM permeability by opening and closing specific pores. It appears that under reducing or mildly oxidizing conditions, when bacteria can grow without extensive damage to intrabacterial components, rapid acquisition of hydrophilic nutrients from the environment requires the wide OmpC pore. Under more oxidizing conditions, oxidative stress can damage bacteria and by limiting the influx through closure of OmpC, bacteria likely protect themselves against ROS. Our data suggest that under these circumstances, bacteria facilitate decreased diffusion over the OM through the much smaller OmpA pore. It is likely that other pores and channels are involved in H_2_O_2_ diffusion, although in this study, we focused on OmpA and OmpC only.

Previous studies by another group have identified OmpD as the main porin that facilitates H_2_O_2_ transport over the OM (30). Although, at first glance, these results appear to contradict our observations, substantial differences in experimental design may explain the different involvements of OMPs in H_2_O_2_ transport. Most notably, different growth media were used for *in vitro* experimentation. Whereas the *Salmonella* growth conditions in our study closely mimic the conditions encountered in the intracellular environment of macrophages (31), previous data were obtained after the growth of bacteria in lysogeny broth (30). In a separate study, Kröger et al. used transcriptome sequencing analysis to show that OmpD and OmpF are severely downregulated under conditions that mimic intracellular growth, while OmpC and OmpA are upregulated (32). On the basis of these results, we conclude that growth conditions severely impact which OMPs are responsible for H_2_O_2_ transport across the OM.

The structure and function of OmpA have been controversial, with some models suggesting switching between protein conformations, resulting in larger and smaller porins (33, 34). Interestingly, some reports indicate that the disulfide bond is crucial for the folding of OmpA in the larger pore conformation (35). These results provide a potential explanation for one underlying mechanism for our observations since abrogation of the disulfide bond leads to lower OM permeability. Alternatively, the periplasmic domain of OmpA has been shown to bind peptidoglycan, bridging peptidoglycan to the OM (36). A different binding affinity for peptidoglycan, which may be regulated by the disulfide bond, could therefore affect the stabilization of the OM and regulate permeability. A more in-depth analysis of OmpA permeability in live bacteria is required to gain full insight into the underlying mechanisms that regulate OmpA permeability.

Although this is the first report of periplasmic proteins and periplasmic protein domains controlling OM permeability for ROS, several OMPs have been reported to directly bind small periplasmic proteins, indicating that these mechanisms may be widespread among Gram-negative bacteria (25, 37, 38). In mitochondria (which are evolutionarily related to bacteria), the concept of a plug protein controlling membrane permeability has already been shown to exist (39). Although we believe our data indicate the involvement of HslT and TrxA in the regulation of OmpC permeability, we have thus far been unable to obtain conclusive evidence of direct binding among OmpC, HslT, and TrxA. It is therefore challenging to speculate on the exact mechanisms that drive closing of the OmpC pore when oxidative stress is encountered. Previously, it has been shown that OmpC has alternative states with different permeabilities (40). HslT and TrxA might influence the proportion of channels in various states and thereby regulate permeability. Real-time monitoring of membrane transport in live bacteria is required for further exploration of OM permeability-controlling mechanisms. GFP biosensors allow for this, and the use of roGFP2 in this study shows the potential of measuring real-time H_2_O_2_ fluctuations to examine membrane transport in live bacteria.

Our results show compelling evidence that increasing OM permeability has great potential for limiting the number of bacteria that survive antibiotic treatment. Recently, it has been shown that macrophage-induced antibiotic tolerance is the same in *phox^−/−^* mutant and WT macrophages (10). Since ROS are not the driving force behind the phenotypic chances that occur because of macrophage interaction and lead to antibiotic tolerance, we did not include *phox^−/−^* mutant macrophages or knockout mice deficient in ROS generation. Our findings that *hslT* mutant bacteria have increased OM permeability suggest that HslT could be targeted to increase membrane permeability and thereby the bacterial killing efficacy of antibiotics.

A recent study suggests that targeting of OM permeability can be an effective strategy for increasing antibiotic efficacy (41). That study showed that treatment of Gram-negative bacteria with a combination of silver and antibiotics increases OM permeability and thereby decreases bacterial survival after antibiotic treatment (41). These results show the promise of silver as a new approach for treating bacterial infections; however, the use of silver is nonspecific and would affect all commensal and symbiotic Gram-negative members of the intestinal microbiota. Recurrent problems with untreatable *Clostridium difficile* infections after harsh antibiotic regimens highlight the importance of a targeted approach to protect a balanced microbiota. The identification of naturally occurring mechanisms that control permeability allows for the design of specific antimicrobials that target these mechanisms. By targeting specific mechanisms, these antimicrobials would be more specific for pathogenic bacteria, thereby eliminating infection while maintaining a healthy, balanced microbiome.

Although we identified two OM pores that were controlled for the ability to facilitate the diffusion of H_2_O_2_, we specifically examined HslT as a potential target for increasing OM permeability instead of targeting the OmpA periplasmic domain. Our reasoning was that antibiotics are often bigger molecules that permeate very slowly (or not at all) through the small OmpA pore (21). Therefore, artificially increasing permeability through the bigger OmpC pore during antibiotic treatment would have a far greater effect on antibiotic killing than opening OmpA. Additionally, the OmpA pore appeared to be open under oxidative stress conditions that are encountered during infection. Thus, we expect that targeting the OmpA periplasmic domain would have only a limited effect.

It is technically difficult to separate defects in bacterial virulence resulting from altered OM permeability from differences in antibiotic tolerance during infection experiments. During *ex vivo* experiments, we were able to experimentally separate the macrophage interaction (virulence phenotype) from the experimentation that analyzed antibiotic killing of bacteria (Fig. 4C). We therefore believe that our *in vitro* data conclusively indicate that increasing OM permeability can be used to increase antibiotic efficacy. Although we attempted to control for colonization/virulence defects during *in vivo* experiments by normalizing to an untreated control (Fig. 4H), it is considerably more challenging to separate attenuation by virulence defects from differences in antibiotic killing. Our results obtained with a mouse model of infection should therefore be interpreted with caution and used as a starting point for further experimentation aimed at increasing bacterial killing by antibiotics.

In conclusion, we identified two OM pores that bacteria can control to rapidly reduce their OM permeability in response to oxidative stress. These mechanisms were critical for bacterial survival under oxidizing conditions, and our results indicate that these mechanisms could be manipulated and become potential targets for increasing antibiotic efficacy. Pores in many OMPs are probably controlled by similar mechanisms that can be targeted by new antimicrobial therapies in order to increase OM permeability and thereby enhance the antimicrobial effects of conventional antibiotics.

## MATERIALS AND METHODS

### Bacterial strains.

All experiments were done with *S*. Typhimurium strain 12023. roGFP2 was cloned from the pRSETB vector (42) into the pfpv25 vector for constitutive expression of roGFP2 and transformed into *S*. Typhimurium (16).

### Gene deletions.

Clean, nonpolar deletions in the HpxF^−^ background and in the WT background were made by allelic exchange with the pCDV442 vector as previously described (43).

### Fluorescence measurement of fluctuations in intrabacterial redox potential.

Real-time fluctuations of the intrabacterial H_2_O_2_ concentration were analyzed in a Tecan fluorescence plate reader with excitation at 405 and 480 nm, while emission was measured at 510 nm (16). Prior to analysis, bacteria were grown to an optical density at 600 nm (OD_600_) of ~1.0 in low-phosphate medium at pH 5.8 (which is used to mimic intracellular conditions). Bacteria were washed and resuspended in saline at an OD_600_ of 2. One hundred microliters of this bacterial culture was loaded into a black, clear-bottom, 96-well plate. Bacteria were challenged with 0, 20, 40, 60, 80, 100, 150, or 200 µM H_2_O_2_, and H_2_O_2_ influx was analyzed. Background signals from nonfluorescent bacteria were obtained in the same experiment. Additionally, the signals for fully oxidized and fully reduced bacteria were obtained by adding 100 mM H_2_O_2_ and 10 mM DTT to the bacterial culture at the start of the experiment. The 405/480-nm ratios were normalized to the 405/480-nm ratios for maximum oxidized and reduced conditions. Each experiment was replicated at least four times. No statistical methods were used to predetermine sample size.

### Calculation of redox potential.

Calculation of the intrabacterial redox potential (E_roGFP2_) was done as previously described (16, 44).

### Calculation of H_2_O_2_ influx.

To determine Δ*C*, which is given by the equation Δ*C* = *C*_out_ − *C*_in_, we obtained the intrabacterial [H_2_O_2_] (*C*_in_) by using the correlation between the normalized 405/480-nm ratio and the intrabacterial [H_2_O_2_] that we obtained in Fig. S1C in the supplemental material. Additionally, by using *k*_cat_ (see Fig. S1B) and by knowing the time that has passed since the initial H_2_O_2_ challenge (*dt*), we calculated *C*_out_ as follows: *C*_out_ = *C*_start_ − (*k*_cat_ ⋅ *dt*).

For most of the analysis in this study, we chose to calculate the influx before the switching point at Δ*C* = 45 µM and after the switching point at Δ*C* = 120 µM. At these moments in our data analysis, we obtained the H_2_O_2_ influx as follows: Influx = (Δ*C*_in_/*dt*).

### Quantification of antibiotic-tolerant bacteria.

Quantification of antibiotic tolerance was done as previously described (10). In short, bacteria were grown overnight in LB medium and inoculated into fresh LB medium (1/400 dilution) containing cefotaxime (100 µg/ml). Prior to treatment, a sample was taken and dilutions were plated to determine the inoculum size. Bacteria were incubated for 24 h at 37°C while shaking before being washed twice in phosphate-buffered saline (PBS) and plated for CFU counting. Macrophage-induced antibiotic tolerance was induced by incubation with BMDM for 30 min. After 30 min, cells were washed with PBS three times and then lysed with lysis buffer (1% Triton X-100, 0.1% SDS in PBS). Inoculum size was determined by plating the bacteria after interaction with BMDM. The inoculum was incubated in fresh LB medium containing cefotaxime (100 µg/ml) for 24 h at 37°C while shaking before being washed twice in PBS and plated for CFU counting. The total number of antibiotic-tolerant bacteria was normalized to the inoculum size.

### Mouse infections.

Six- to 8-week-old C57BL/6 female mice were purchased from Jackson Laboratories. Twenty-four hours prior to infection, mice were given 20 mg of streptomycin sulfate (Gold Biotechnology) by oral gavage to ensure high levels of *Salmonella* gastrointestinal colonization*.* Mice were orally infected with ~5 × 10^7^ CFU of streptomycin-resistant *S*. Typhimurium strain 12023 (WT or *hslT* or *ompC hslT* mutant). Since mice were infected with different bacterial strains (and mice shed infectious bacteria after infection), mice infected with different strains were kept in different cages. Beginning 24 h following infection, mice were administered either 100 mg/kg cefotaxime in H_2_O (Cayman Chemical Co.) by subcutaneous injection or control H_2_O subcutaneous injections twice daily for 2 days. Mice were sacrificed 3 days after *S*. Typhimurium infection, and organs were collected, weighed, homogenized, and plated on LB plates containing 100 µg/ml streptomycin sulfate (Gold Biotechnology) for determination of numbers of *S*. Typhimurium CFU per gram of tissue. A blinded observer determined CFU counts. The animal work presented in this report was approved by the University of British Columbia Animal Care Committee (certificate number A13-0265). Results presented in this report show data from individual mice tested. The data shown are pooled data from at least two different experiments. No statistical methods were used to predetermine sample size.

## SUPPLEMENTAL MATERIAL

Text S1  Detailed materials and methods. Download Text S1, DOCX file, 0.04 MB

Figure S1  Experimental values for calculations of OM permeability and influx. (A, B) Catalytic activity of WT and HpxF^−^ *S*. Typhimurium detoxifying H_2_O_2_. Catalytic activity was calculated by dividing the time it takes to completely eradicate the H_2_O_2_ challenge by the average [H_2_O_2_]. (C) Correlation between the intrabacterial [H_2_O_2_] (µM) and the normalized 405/480-nm ratio for the HpxF^−^ strain. (D) H_2_O_2_ influx in stationary HpxF^−^ *S*. Typhimurium before the switching point. (E) Comparison of H_2_O_2_ influx levels in stationary and log-phase HpxF^−^ *S*. Typhimurium before the switching point. (F) Comparison of H_2_O_2_ influx levels in stationary- and log-phase HpxF^−^ *S*. Typhimurium after the switching point. (G) Comparison of real-time H_2_O_2_ influx levels in spheroplasts (HpxF^−^ *S*. Typhimurium without OMs) and HpxF^−^ *S*. Typhimurium with an OM. Each value represents the average of four separate experiments. Download Figure S1, TIF file, 0.7 MB

Figure S2  Control for equal expression of OmpA in different mutants. Western blot assay showing expression of OmpA in different mutants. Equal expression of OmpA is observed in all of the mutants, except the *ompA* and *ompA*^C-term^ mutants, in which the *ompA* gene has been deleted or partially deleted, respectively. A loading control with an antibody for DnaK shows that the same amount of total protein was loaded into each well. Download Figure S2, TIF file, 1.6 MB

Figure S3  Colonization of mice by WT and *ompC*, *hslT*, and *ompC hslT* mutant *S*. Typhimurium at 3 days postinfection without antibiotic treatment. (A) Mice were infected with WT or *ompC*, *hslT*, or *ompC hslT* mutant bacteria, and colonization of the spleen, liver, MLN, cecum, and colon was analyzed by *S*. Typhimurium CFU counting at 3 days postinfection. Only for *ompC* mutant bacteria did we find less colonization of the spleen, liver, and MLN than by WT bacteria. (B) CFU counts of WT and *ompC*, *hslT*, and *ompC hslT* mutant bacteria that survived antibiotic treatment in the spleen, liver, and MLN at 3 days postinfection (p.i.) and subsequent treatment with cefotaxime. In all organs of mice infected with *hslT* mutant bacteria, significantly lower numbers of bacteria were found than in mice infected with WT *S*. Typhimurium. CFU counts of *hslT* mutant *S*. Typhimurium in the liver and MLN after treatment were significantly lower than those of *ompC* or *ompC hslT* mutant bacteria. All experiments were done with mutants in the WT background. Statistical significance was determined by a Kruskal-Wallis test with comparison to the WT control group (*, *P* < 0.05; **, *P* < 0.01; ***, *P* < 0.001; ****, *P* < 0.0001; ns, not significant). Download Figure S3, TIF file, 1 MB

Table S1  Peptides identified in a SILAC mass spectrometry experiment for identification of reversible disulfide bonds in the *S*. Typhimurium proteome. Disulfide bond formation in the *S*. Typhimurium proteome was examined under oxidizing and normoxic conditions. Oxidative stress was induced by adding H_2_O_2_ or nitric oxide (spermine NONOate). Peptides containing cysteines that were involved in disulfide bonds were enriched, and the abundance ratio under oxidizing/normoxic conditions is presented. Experiments were performed in triplicate. Table S1, DOCX file, 0.02 MB
