# Supplementary material for: Salmonella Rapidly Regulates Membrane Permeability To Survive Oxidative Stress
Source: mBio. 2016 Aug 9;7(4):e01238-16. doi: 10.1128/mBio.01238-16 (PMC4992977; doi:10.1128/mBio.01238-16)
Supplement: Text S1 — Detailed materials and methods. Download [file mbo004162943s1.docx]

Supplementary Material for

***Salmonella*** **rapidly regulates membrane permeability to survive oxidative stress.**

**Joris van der Heijden, Lisa A. Reynolds, Wanyin Deng, Allan Mills, Roland Scholz, Koshi Imami, Leonard J. Foster, Franck Duong, B. Brett Finlay^*^**

*Corresponding author. E-mail: [bfinlay@interchange.ubc.ca](mailto:bfinlay@interchange.ubc.ca).

**This PDF file includes:**

Supplemental Experimental Procedures

Figs. S1, S2, S3 and Table 1.

**Material and methods**

**Bacterial strains**

All experiments were done using the *S.* Typhimurium strain 12023. roGFP2 was cloned into the pfpv25 vector for constitutive expression of roGFP2 and transformed into *S.* Typhimurium*.* After transformation 50 μg/ml carbenicillin was added to the growth media to maintain the plasmid. The HpxF^-^ strain was kindly donated to us by Dr. Laurent Aussel ([*1*](#_ENREF_1)). The point mutations in *ompA^C327S^*, *trxA^C33S^* and *trxA^C33SC36S^* were made using the QuikChange® II Site-Directed Mutagenesis Kit (Stratagene).

**Gene deletions**

Clean deletions in the HpxF^-^ background and in the WT background of *S.* Typhimurium were made using allelic exchange as previously described ([*2*](#_ENREF_2)). In short, 500 base pair flanking regions surrounding, but generating an in-frame deletion of the gene of interest, were amplified by overlapping PCR and cloned into the pCDV442 vector. After verification by sequencing, this vector was transformed into the SM10λpir strain while growth media were supplemented with 50 μg/ml DAP (Diaminopimelic acid) and conjugated into recipient *S.* Typhimurium HpxF^-^ or subsequent target strains. Plasmid integration was screened by antibiotic selection, and subsequent plasmid excision was driven by sucrose counter selection and verified by antibiotic sensitivity. Gene deletions or chromosomal point mutations were verified by PCR and sequencing.

**Complementation experiments**

The pBAD33 plasmid containing the gene of interest was transformed into the deletion strain and maintained by addition of 30 μg/ml chloramphenicol. In the 5 hours prior to membrane permeability experiments, no chloramphenicol was added and instead 0.2% arabinose was added to the growth media.

**Native acrylamide gel electrophoresis**

The OmpA periplasmic domain and the OmpA^C327S^ mutant periplasmic domain (AA198-350) from *S.* Typhimurium SL1344 were cloned into the pET-28a vector (N-terminal HIS-tag) and transformed into BL21(DE3) bacteria. 50 mL of cultures were grown to OD~1.0 after which protein expression was induced by adding 0.1mM IPTG. The temperature was lowered to 16 °C and the cultures were incubated overnight.

After overnight expression, bacteria were spun down at 3500 x g and washed with 50 mL saline solution. Bacteria were lysed by sonication in lysis buffer (20mM HEPES, 20mM imidazole, 120 mM Potassium acetate, 0.1% Triton X100, 10 µg/ml DNase, 10 µg/ml RNase, Protease inhibitor cocktail, pH 7.5) and the proteins were purified by loading them onto a Ni-column. 20 μg of protein was pre-treated with varying concentrations of hydrogen peroxide or DTT for 60 minutes prior to loading the protein onto a 15% native acrylamide gel. The native gel was analyzed by Coomassie Blue staining.

**Western blot**

WT, *ompA*, *ompA^198-350^, ompA^C327S^, ompC, hslT* and *ompChslT* bacteria were grown for 5 hours. From each culture a similar amount of total protein was loaded onto a SDS-PAGE gel. After running the SDS-PAGE gel, the proteins were transferred to a nitrocellulose membrane and the level of OmpA expression was analyzed by using a rabbit anti-OmpA polyclonal antibody (MyBiosource, #MBS1488520). A loading control was included by using a mouse anti-DnaK antibody.

**Stable Isotope Labeling by Amino Acids (SILAC) proteomics**

A *Salmonella* strain carrying deletions in *lysA* and *argH* was grown in LPM. In this growth media, casamino acids were replaced by addition of individual amino acids at the following concentrations: Ala, 2.8%; Asp, 6.3%; Cys, 0.3%; Glu, 21.1%; Gly, 2.2%; His, 2.7%; Ile, 5.6%; Leu, 8.4%; Met, 2.7%; Phe, 4.6%; Pro, 9.9%; Ser, 5.6%; Thr, 4.2%; Trp, 1.1%; Tyr, 6.1%; Val, 5.0%; Arg, 3.6%; and Lys, 7.5%). In order to label bacterial proteins with heavy amino acids, the Lys and Arg residues were replaced by [4,4,5,5-D4]lysine and [U-13C6]arginine instead of natural abundance, isotopically labeled arginine and lysine.

**Identifying proteins with reversible disulfide bonds**

Light and heavy labelled cultures were grown for 5 hours at 37°C. 5 ml of the heavy labelled culture was then subjected to a challenge with either 2 x 5 mM H_2_O_2_ (separated by a 10 minute interval) or a challenge with 5mg/ml of SpermineNONOate, whereas 5 ml of the light labelled bacterial culture was left untouched. After 20 minutes the cultures were spun down, resuspended in Guanidine/MOPS buffer (6M Guanidine HCL pH7.5, 150mM NaCl, 5mM EDTA, 25mM MOPS) and lysed by sonication. Alkylation of free thiol groups was done by adding 40mM N-ethyl maleimide for two hours at room temperature. After this, 4 volumes of ice cold methanol were added and the protein fraction was incubated overnight at -20 °C. The next morning, protein fractions were spun down for 45min at 15 000 x g at 4°C, and the protein pellet was washed twice with ice cold methanol. The protein pellets were dissolved in 100 μL of the Guanidine/MOPS buffer and protein fractions were pooled. 10mM DTT was added at 37 °C for 30 min prior to addition of 0.4mM HPDP-biotin (Proteochem). Samples were mixed and incubated for 2 hours at room temperature. After labelling with HPDP-biotin, LysC was added and protein fractions were incubated for 3 hours at 37°C followed by a 4x dilution in 50mM ammonium bicarbonate, and then trypsin was added to incubate overnight. After the tryptic digest was complete we removed excess HPDP-biotin. To do this, the HPDP-biotin labeled peptides were desalted with C18 STAGE tips and after that we enriched for HPDP-biotin peptides using Neutravidin beads (Fisher Scientific) that had previously been equilibrated with 20 mM Ammonium bicarbonate. Samples were left on rotation for 1 h at room temperature to allow binding of the biotinylated peptides to NeutrAvidin. After centrifugation at 200 × g for 10 s, the supernatant was discarded and the beads were washed 4 times with 20 mM ammonium bicarbonate in 0.5 M NaCl solution. Two additional washings with 20% acetonitrile in 5 mM ammonium bicarbonate were done to remove nonspecifically bound peptides. Peptides were eluted by incubating the beads for 10 min with 2 volumes of 100 mM mercaptoethanol. The samples were then dried in a speedvac and resuspended in 20 μL of 5% formic acid and cleaned using C18 Zip Tips prior to mass spectrometric analysis.

**NanoLC-MS/MS analysis**

NanoLC-MS/MS analysis was conducted using an LTQ-Orbitrap Velos mass spectrometer (Thermo Fisher Scientific) equipped with an 1100 series nanoflow HPLC system (Agilent) as described (*6*). An in-house analytical column (200 mm length x 75 μm inner diameter) was prepared with ReproSil-Pur C18-AQ materials (3 μm, Dr. Maisch). Prior to analytical separation, peptides were loaded for 10 min onto a fused 5 silica trap column (20 mm length x 100 μm inner diameter), which was packed with 5 μm Aqua C18 beads (Phenomenex). Analytical separation was performed at a flow rate of 5 μL/min. A 90 min gradient from 100% buffer A (0.5% acetic acid) to 40% buffer B (0.5% acetic acid, 80% acetonitrile) was performed at a flow rate of 250 nL/min to elute the peptides. The following settings for MS analysis were chosen: one full scan (resolution 60,000; m/z 300–1,500) followed by top 10 MS/MS scans using CID in the linear ion trap (min. signal required, 200; isolation width, 3; normalized collision energy, 35; activation Q, 0.25; activation time, 10 ms) using dynamic exclusion (repeat count, 1; repeat duration, 30 s; exclusion list size, 500; exclusion duration, 60 s).

**Mass spectrometry data analysis**

MaxQuant (v1.3.0.5) (*7*) was used to analyze all raw data. For the search the following parameters were included: two missing cleavage sites, cysteine carbamidomethyl fixed modification, as well as variable modifications, such as methionine oxidation and N-terminal protein acetylation. MS/MS tolerance was set to 0.5 Da. Peptide mass tolerance was 6 ppm. Andromeda (*8*) was used to search against the UniProt/Swiss-Prot *Salmonella* Typhimurium database (downloaded on July 2011) including common serum contaminants and enzyme sequences. 1% was set as the false discovery rate (FDR) at peptide and at protein level.

**Culturing bone marrow derived macrophages**

Bone marrow was collected from the tibias and femurs of 6-8 week old WT C57BL/6J, iNOS-/- (B6.129P2-Nos2tm1Lau/J) and gp91phox-/- (B6.129S6-Cybbtm1Din/J) male mice (all purchased from Jackson Laboratory). In each well of a 6-well plate, 1x10^6^ cells were seeded in growth media (RPMI 1640 containing L-Glutamine; Life Technologies, supplemented with 10% heat-inactivated FBS; Life Technologies, 100 U/ml Penicillin and 100 μg/ml Streptomycin; Life Technologies and 20 ng/ml M-CSF; Peprotech). Cells were incubated at 37°C with 5% CO2 for 7 days. On days 4 and 6, media was removed and replaced with 2 ml fresh medium. On day 7, culture supernatant containing non-adherent cells were removed prior to performing assays. The purity of adherent cells was confirmed by flow cytometry for all genotypes on day 7 of culture, and >97% of cells were CD45+CD11b+F4/80+.

**References**

1. M. Hebrard, J. P. Viala, S. Meresse, F. Barras, L. Aussel, *J Bacteriol* **191**, 4605-4614 (2009).

2. R. A. Edwards, L. H. Keller, D. M. Schifferli, *Gene* **207**, 149-157 (1998).
