# Supplementary figures and images for: Salmonella Rapidly Regulates Membrane Permeability To Survive Oxidative Stress
Source: mBio. 2016 Aug 9;7(4):e01238-16. doi: 10.1128/mBio.01238-16 (PMC4992977; doi:10.1128/mBio.01238-16)

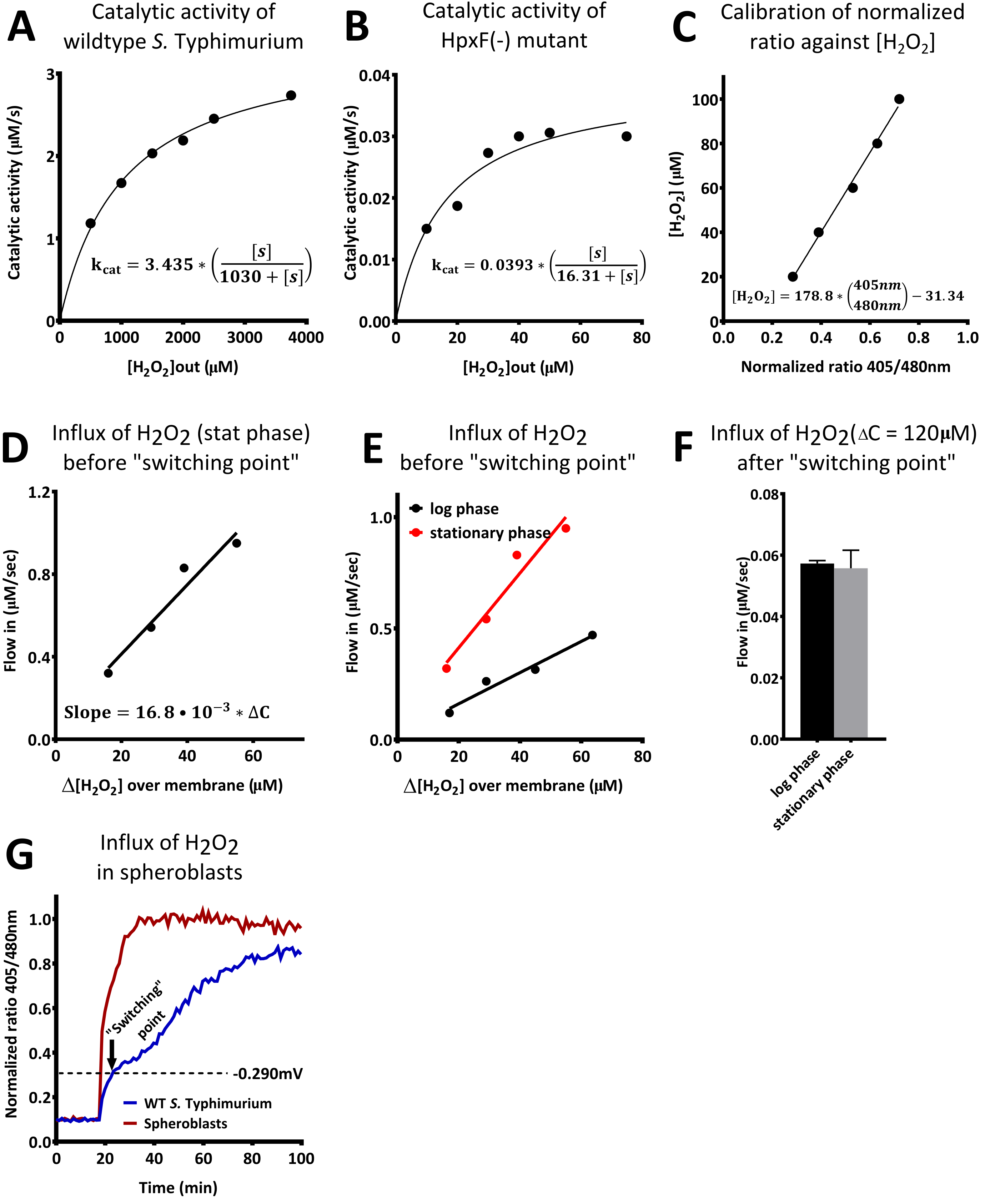

Supplement: Figure S1 — Experimental values for calculations of OM permeability and influx. (A, B) Catalytic activity of WT and HpxF− S. Typhimurium detoxifying H2O2. Catalytic activity was calculated by dividing the time it takes to completely eradicate the H2O2 challenge by the average [H2O2]. (C) Correlation between the intrabacterial [H2O2] (µM) and the normalized 405/480-nm ratio for the HpxF− strain. (D) H2O2 influx in stationary HpxF− S. Typhimurium before the switching point. (E) Comparison of H2O2 influx levels in stationary and log-phase HpxF− S. Typhimurium before the switching point. (F) Comparison of H2O2 influx levels in stationary- and log-phase HpxF− S. Typhimurium after the switching point. (G) Comparison of real-time H2O2 influx levels in spheroplasts (HpxF− S. Typhimurium without OMs) and HpxF− S. Typhimurium with an OM. Each value represents the average of four separate experiments. Download [file mbo004162943sf1.tif]

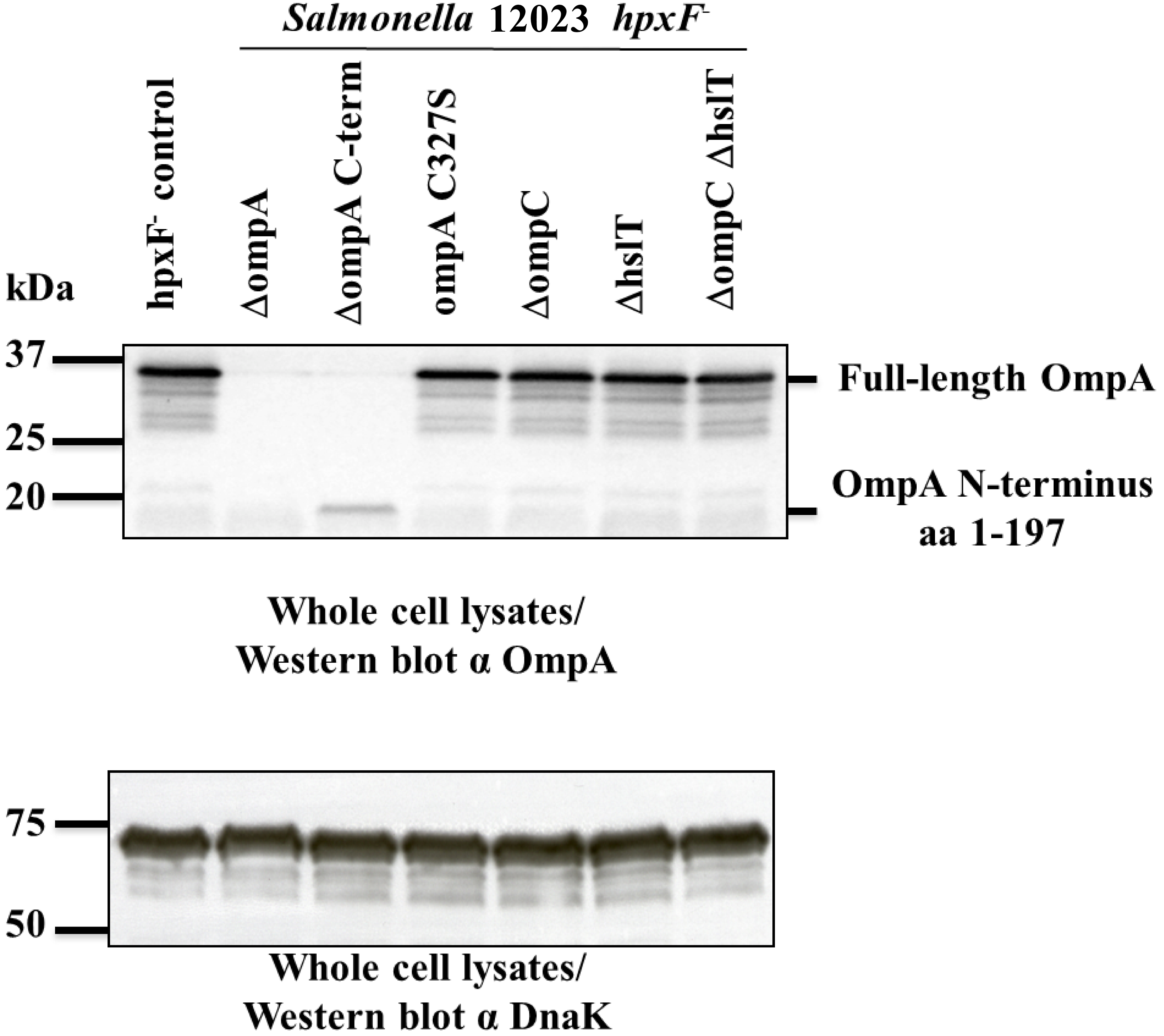

Supplement: Figure S2 — Control for equal expression of OmpA in different mutants. Western blot assay showing expression of OmpA in different mutants. Equal expression of OmpA is observed in all of the mutants, except the ompA and ompAC-term mutants, in which the ompA gene has been deleted or partially deleted, respectively. A loading control with an antibody for DnaK shows that the same amount of total protein was loaded into each well. Download [file mbo004162943sf2.tif]

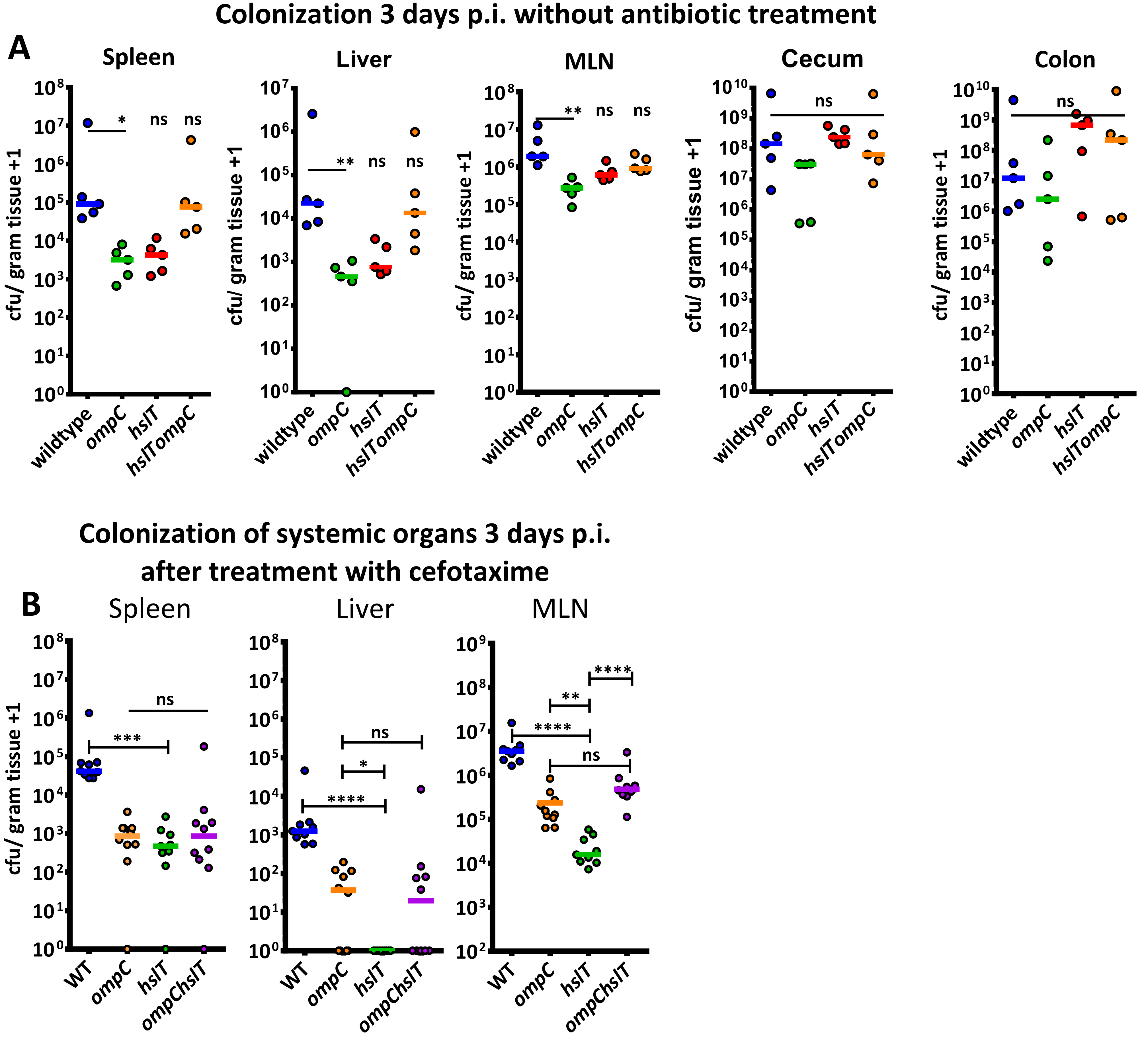

Supplement: Figure S3 — Colonization of mice by WT and ompC, hslT, and ompC hslT mutant S. Typhimurium at 3 days postinfection without antibiotic treatment. (A) Mice were infected with WT or ompC, hslT, or ompC hslT mutant bacteria, and colonization of the spleen, liver, MLN, cecum, and colon was analyzed by S. Typhimurium CFU counting at 3 days postinfection. Only for ompC mutant bacteria did we find less colonization of the spleen, liver, and MLN than by WT bacteria. (B) CFU counts of WT and ompC, hslT, and ompC hslT mutant bacteria that survived antibiotic treatment in the spleen, liver, and MLN at 3 days postinfection (p.i.) and subsequent treatment with cefotaxime. In all organs of mice infected with hslT mutant bacteria, significantly lower numbers of bacteria were found than in mice infected with WT S. Typhimurium. CFU counts of hslT mutant S. Typhimurium in the liver and MLN after treatment were significantly lower than those of ompC or ompC hslT mutant bacteria. All experiments were done with mutants in the WT background. Statistical significance was determined by a Kruskal-Wallis test with comparison to the WT control group (*, P < 0.05; **, P < 0.01; ***, P < 0.001; ****, P < 0.0001; ns, not significant). Download [file mbo004162943sf3.tif]
