# Supplementary material for: Salmonella Rapidly Regulates Membrane Permeability To Survive Oxidative Stress
Source: mBio. 2016 Aug 9;7(4):e01238-16. doi: 10.1128/mBio.01238-16 (PMC4992977; doi:10.1128/mBio.01238-16)
Supplement: Table S1 — Peptides identified in a SILAC mass spectrometry experiment for identification of reversible disulfide bonds in the S. Typhimurium proteome. Disulfide bond formation in the S. Typhimurium proteome was examined under oxidizing and normoxic conditions. Oxidative stress was induced by adding H2O2 or nitric oxide (spermine NONOate). Peptides containing cysteines that were involved in disulfide bonds were enriched, and the abundance ratio under oxidizing/normoxic conditions is presented. Experiments were performed in triplicate. [file mbo004162943st1.docx]

| **Row Labels** | **Row Labels2** | **H2O2_1** | **H2O2_2** | **H2O2_3** | **NO_1** | **NO_2** | **NO_3** |
| --- | --- | --- | --- | --- | --- | --- | --- |
| **sp\|P02936\|OMPA_SALTY Outer membrane protein A OS=Salmonella typhimurium GN=ompA PE=3 SV=2** | **_AALIDCLAPDR_** |  | **0.0443** |  |  |  | **0.7530** |
| **sp\|P02936\|OMPA_SALTY Outer membrane protein A OS=Salmonella typhimurium GN=ompA PE=3 SV=2** | **_AALIDCLAPDRR_** | **0.77027** | **0.9490** |  |  |  | **0.63542** |
| sp\|Q8ZK71\|MSRA_SALTY Peptide methionine sulfoxide reductase MsrA OS=Salmonella typhimurium GN=msrA PE=3 SV=1 | _EVCSGQTGHAEAVR_ |  |  |  | 0.01916 |  |  |
| sp\|P66131\|RL27_SALTY 50S ribosomal protein L27 OS=Salmonella typhimurium GN=rpmA PE=3 SV=2 | _FHAGTNVGCGR_ |  |  |  |  | 0.032623 |  |
| sp\|P0A7S6\|RS12_SALTY 30S ribosomal protein S12 OS=Salmonella typhimurium GN=rpsL PE=3 SV=2 | _GALDCSGVKDRK_ |  | 0.77901 |  | 0.50734 |  |  |
| **sp\|P02936\|OMPA_SALTY Outer membrane protein A OS=Salmonella typhimurium GN=ompA PE=3 SV=2** | **_GM(ox)GESNPVTGNTCDNVKPR_** |  |  |  | **0.20515** |  | **0.71868** |
| **sp\|P02936\|OMPA_SALTY Outer membrane protein A OS=Salmonella typhimurium GN=ompA PE=3 SV=2** | **_GMGESNPVTGNTCDNVKPR_** | **0.65918** | **0.66305** | **0.33121** | **0.30562** | **0.44263** | **0.67309** |
| sp\|P0A1H5\|EFTU_SALTY Elongation factor Tu OS=Salmonella typhimurium GN=tufA PE=3 SV=2 | _HYAHVDCPGHADYVK_ |  | 0.07256 | 0.03816 | 0.07291 | 0.034922 | 1.1811 |
| sp\|P0A2R4\|RPOC_SALTY DNA-directed RNA polymerase subunit beta OS=Salmonella typhimurium GN=rpoC PE=3 SV=1 | _LHQCGLPK_ |  |  |  | 0.09865 |  |  |
| sp\|P74881\|PUR4_SALTY Phosphoribosylformylglycinamidine synthase OS=Salmonella typhimurium GN=purL PE=1 SV=3 | _RCQEVIDR_ |  |  |  | 0.48189 |  |  |
| sp\|P0A1H5\|EFTU_SALTY Elongation factor Tu OS=Salmonella typhimurium GN=tufA PE=3 SV=2 | _STCTGVEMFRK_ |  |  |  | 0.25749 |  | 1.1347 |
| sp\|P64052\|EFTS_SALTY Elongation factor Ts OS=Salmonella typhimurium GN=tsf PE=3 SV=1 | _TGAGMMDCKK_ |  | 0.67403 |  | 0.3769 |  |  |
